# Supplementary material for: Identification of photoperiod-induced specific miRNAs in the adrenal glands of Sunite sheep (Ovis aries)
Source: Front Vet Sci. 2022 Jul 22;9:888207. doi: 10.3389/fvets.2022.888207 (PMC9354845; doi:10.3389/fvets.2022.888207)
Supplement: Supplementary file 1 [file Data_Sheet_1.ZIP › Supplementary file/miRDeep2 file.zip!/miRDeep2 file/Novel_Structure/LP42A1/Novel_267.pdf]

Provisional ID : NC_019460.2_7144
Score total : 1.4
Score for star read(s) : -1.3
Score for read counts : 0
Score for mfe : 1.1
Score for randfold : 1.6
Score for cons. seed :
Total read count : 16
Mature read count : 16
Loop read count : 0
Star read count : 0

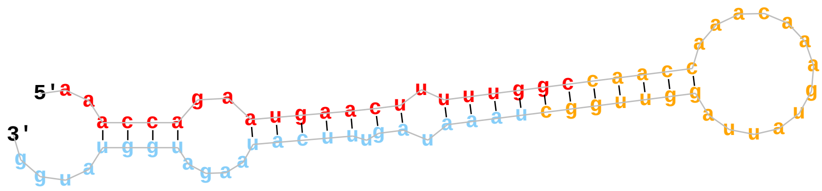

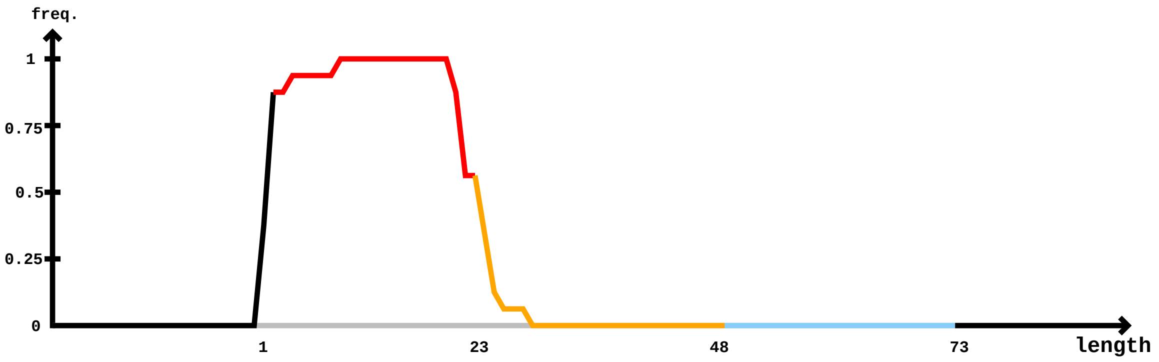


```
                     Mature                                           Star
5'- guuucauaagaugguacgggcaaaccagaaugaacuuuuuggccaaccaaacaaaguauuagguuggcuaaauaguuucauaagaugguaugggcaaaccagaaugaacu   -3'    exp
    ..((((....((((.....((.((((..(((((((.(((((((((((.............))))))))))).)).)))))....)))).)).....))))..)))))..   reads      mm        sample
    ....................caaaccagaaugaacuuuuu......................................................................   1          0         seq
    ....................Aaaaccagaaugaacuuuuug.....................................................................   5          1         seq
    .....................aaaccagaaCgaacuuuuu......................................................................   1          1         seq
    .....................aaaccCgaaugaacuuuuuggc...................................................................   7          1         seq
    .......................acUagaaugaacuuuuuggcca.................................................................   1          1         seq
    ............................aaugaacuuuuuggccaacc..............................................................   1          0         seq
```
